# Compounding MgCl_2_·6H_2_O with NH_4_Al(SO_4_)_2_·12H_2_O or KAl(SO_4_)_2_·12H_2_O to Obtain Binary Hydrated Salts as High-Performance Phase Change Materials

**DOI:** 10.3390/molecules24020363

**Published:** 2019-01-21

**Authors:** Wanchun Sun, Yan Zhou, Jinxin Feng, Xiaoming Fang, Ziye Ling, Zhengguo Zhang

**Affiliations:** 1 Key Laboratory of Enhanced Heat Transfer and Energy Conservation, The Ministry of Education, School of Chemistry and Chemical Engineering, South China University of Technology, Guangzhou 510640, China; greycici1114@gmail.com (W.S.); zhouyanscut@163.com (Y.Z.); cefjxfeng@mail.scut.edu.cn (J.F.); cexmfang@scut.edu.cn (X.F.); 2 Guangdong Engineering Technology Research Center of Efficient Heat Storage and Application, South China University of Technology, Guangzhou 510640, China

**Keywords:** latent heat storage, phase change material, magnesium chloride hexahydrate, aluminum ammonium sulfate dodecahydrate, aluminum potassium sulfate dodecahydrate

## Abstract

Developing phase change materials (PCMs) with suitable phase change temperatures and high latent heat is of great significance for accelerating the development of latent heat storage technology to be applied in solar water heating (SWH) systems. The phase change performances of two mixtures, NH_4_Al(SO_4_)_2_·12H_2_O-MgCl_2_·6H_2_O (mixture-A) and KAl(SO_4_)_2_·12H_2_O-MgCl_2_·6H_2_O (mixture-B), were investigated in this paper. Based on the DSC results, the optimum contents of MgCl_2_·6H_2_O in mixture-A and mixture-B were determined to be 30 wt%. It is found that the melting points of mixture-A (30 wt% MgCl_2_·6H_2_O) and mixture-B (30 wt% MgCl_2_·6H_2_O) are 64.15 °C and 60.15 °C, respectively, which are suitable for SWH systems. Moreover, two mixtures have high latent heat of up to 192.1 kJ/kg and 198.1 kJ/kg as well as exhibit little supercooling. After 200 cycles heating-cooling experiments, the deviations in melting point and melting enthalpy of mixture-A are only 1.51% and 1.20%, respectively. Furthermore, the XRD patterns before and after the cycling experiments show that mixture-A possesses good structure stability. These excellent thermal characteristics make mixture-A show great potential for SWH systems.

## 1. Introduction

Thermal energy storage (TES) is a key technology for improving energy efficiency and developing solar energy, since it can solve the mismatch between heat supply and demand in space and time [1,2,3]. Sensible thermal energy storage (STES), latent thermal energy storage (LTES) and thermo-chemical heat storage (TCHS) are the main approaches to realize TES [4]. Compared to STES and TCHS, LTES using phase change materials (PCMs) has attracted increasing interest because of high energy storage density [5]. PCM can absorb or release a large amount of heat during the phase change process while maintaining the system at a constant temperature around its melting point [6]. In order to further enhance the heat storage performance of LTES, PCM should possess the merits of large latent heat, good thermal stability and excellent thermal reliability. Moreover, it is desirable in industrial applications that PCM has a low cost and a suitable phase change temperature for target thermal system. Therefore, the exploration of PCMs that meet the above requirements is of great significance for accelerating the latent heat storage technology to be applied into various fields.

In general, PCMs can be classified into two types: organics and inorganics. Organic PCMs, such as paraffin waxes [7], fatty acids [8], and fatty alcohols [9], have the advantages of little supercooling, no phase separation and non-corrosiveness [10]. However, low thermal conductivity, relatively low phase change enthalpy, large volume change during phase transition and high price limit the widespread use of organic PCMs [11]. By comparison, inorganic PCMs have the advantages of low cost, incombustibility, large latent heat and relatively high thermal conductivity, making them show great potential for application [12]. Inorganic PCMs can be further classified into hydrated salts, molten salts and metals, which are suitable for use in low, medium and high-temperature application fields [13,14]. As a major PCM used in low-temperature environment, hydrated salts show great potential uses in various fields, such as solar water heating (SWH) systems [15], heating and cooling buildings [16], off-peak power utilization [17], heat pump water heating systems, etc. [18]. Nevertheless, the supercooling phenomenon, phase separation and poor thermal reliability of hydrated salts have become unavoidable problems that needs to be solved urgently. For the better applications of hydrated salts, it is necessary to develop an inorganic PCM with high latent heat, good thermal stability and thermal reliability, and low supercooling degree.

In recent years, enormous amounts of research have been carried out on solutions to the disadvantages of hydrated salts. In order to reduce the supercooling degree, the study on the nucleating agents for different hydrated salts has been strongly promoted. Lane [19] conducted experiments to evaluate different nucleating agents for calcium chloride hexahydrate (CaCl_2_·6H_2_O). The experimental results showed that BaI_2_·6H_2_O, SrCl_2_·6H_2_O, and SrBr_2_·6H_2_O could effectively suppress the supercooling of CaCl_2_·6H_2_O. As for sodium acetate trihydrate, the introduction of aluminum nitride [20], silver nanoparticles [21], Si_3_N_4_, ZrB_2_ and SiO_2_ [22] were all proven to be able to effectively reduce the supercooling degree. On the other hand, the phase separation of hydrated salts can be the solved by adding thickeners, such as hydroxyethyl cellulose (HEC) [19,23] and carboxymethylcellulose sodium (CMC) [20,24,25,26]. Furthermore, the combination of porous carriers and hydrated salts can mitigate the negative effects of the phase separation and improve the thermal reliability of the hydrated salts. Due to the large specific surface area, expanded graphite [27,28], expanded perlite [29,30] and diatomite [31,32] were widely used for the preparation of stable composites. In addition to the addition of nucleating agents and thickeners and the combination with porous carriers, a mixture composed of two or more hydrated salts have attracted the attention of more and more scholars. In some hydrated salt systems, mixing with other hydrated salts can not only adjust the melting point, but also reduce the supercooling degree and mitigate phase separation. Nagano et al. [33] added 20 wt% MgCl_2_·6H_2_O to MgNO_3_·6H_2_O to obtain a eutectic mixture. They reported that the mixture exhibited a melting point of about 60 °C, which was much lower than those of two components and was suitable for recovering waste heat. Moreover, Li et al. [23] investigated the CaCl_2_·6H_2_O-MgCl_2_·6H_2_O salt system with various mass fraction of MgCl_2_·6H_2_O. It was found that the mixing of two hydrated salts not only reduced the melting point of CaCl_2_·6H_2_O but also retained most of the melting enthalpy. Note that hydrated salt mixtures usually exhibit different phase change temperatures and latent heat values from those of their components [34]. Therefore, exploration of mixtures is an effective route for developing novel hydrated salt PCMs. According to the requirements of SWH systems, such as suitable melting point, low cost and excellent performance, the mixtures with high latent heat and appropriate phase change temperatures are highly desirable.

In the current work, two hydrated mixtures were explored by combining aluminum ammonium sulfate dodecahydrate (NH_4_Al(SO_4_)_2_·12H_2_O) or aluminum potassium sulfate dodecahydrate (KAl(SO_4_)_2_·12H_2_O) with magnesium chloride hexahydrate (MgCl_2_·6H_2_O). MgCl_2_·6H_2_O is a hydrated salt with a melting point of 117 °C and a melting enthalpy of about 150 kJ/kg. Owing to the abundant magnesium chloride resources in salt lakes, MgCl_2_·6H_2_O is an ideal low-cost PCM. But the high melting point (117 °C) influences the thermal stability and reliability of MgCl_2_·6H_2_O because it is higher than the boiling point of water (100 °C, at standard atmospheric pressure). On the other hand, NH_4_Al(SO_4_)_2_·12H_2_O and KAl(SO_4_)_2_·12H_2_O are common hydrated salts in daily life with phase change temperatures of about 93 °C and latent heat of up to 260 kJ/kg. However, the large supercooling degree (10–15 °C) and relatively high phase change temperature limit their practical application in SWH systems. In order to reduce the melting point of NH_4_Al(SO_4_)_2_·12H_2_O or KAl(SO_4_)_2_·12H_2_O as well as retain the high melting enthalpy, two series of mixtures consisting of MgCl_2_·6H_2_O and NH_4_Al(SO_4_)_2_·12H_2_O or KAl(SO_4_)_2_·12H_2_O were prepared by a melt blending method. The introduction of MgCl_2_·6H_2_O was expected to adjust the thermal properties of NH_4_Al(SO_4_)_2_·12H_2_O or KAl(SO_4_)_2_·12H_2_O to obtain a mixture that meet the requirements of ideal PCM. For determining the compositions of mixtures, the influences of the mass fraction of MgCl_2_·6H_2_O were studied. Furthermore, the obtained mixtures were characterized by various techniques, and their thermal reliabilities were evaluated to provide better guidance for industrial applications.

## 2. Results and Discussion

### 2.1. Determining the Mixture Composition

**Figure 1:**
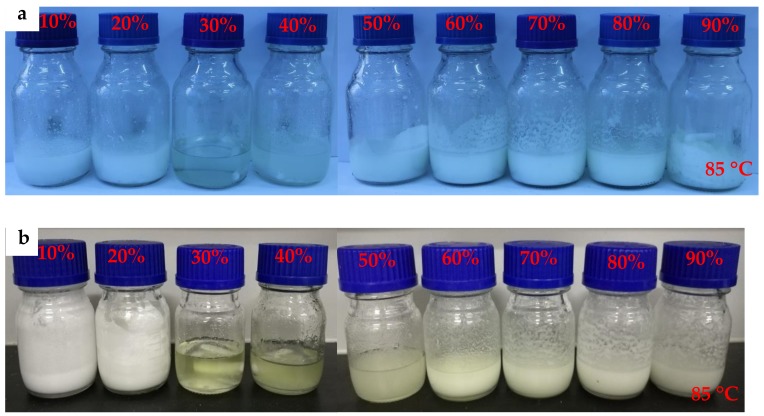
Photographs of (**a**) mixtures-A and (**b**) mixtures-B with different F_MgCl2·6H2O_.

**Figure 2:**
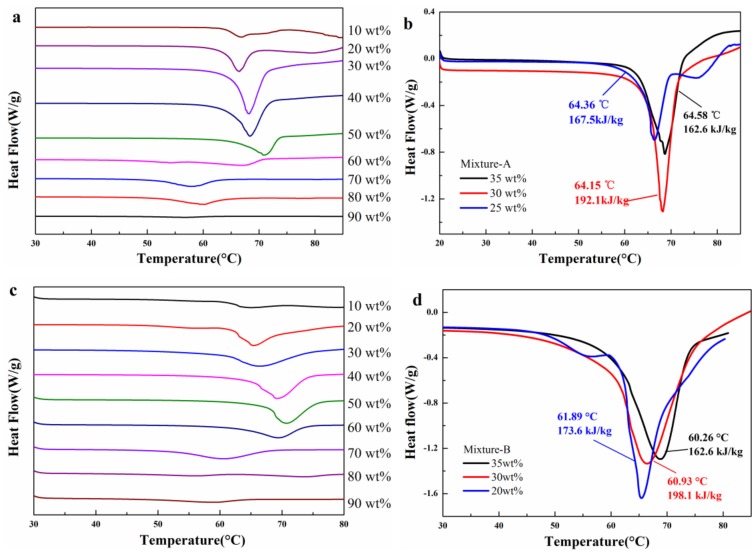
DSC curves of (**a**,**b**) mixtures-A and (**c**,**d**) mixtures-B with different F_MgCl2·6H2O_.

In order to describe the experimental results clearly, the mixtures were classified into 2 series, as shown in Table 1. Figure 1 shows the molten mixtures including mixtures-A and mixtures-B with different mass fractions of MgCl_2_·6H_2_O (F_MgCl2·6H2O_). It can be seen from Figure 1a that mixtures-A containing 30% and 40% MgCl_2_·6H_2_O show transparent and semitransparent solutions, while others are in solid state. On the other hand, mixtures-B containing 30%, 40% and 50% MgCl_2_·6H_2_O are semitransparent solutions, as shown in Figure 1b. To investigate the effect of F_MgCl2·6H2O_ on the thermal properties of mixture-A/mixtures-B, the melting point (T_melt_) and enthalpy (△H) of each sample was measured by DSC (as shown in Figure 2a,c)). The thermal characteristics of mixtures-A/mixtures-B are listed in Table 2. It can been seen that T_melt_ first increases then decreases with the increase of F_MgCl2·6H2O_. As for mixture-A/ mixture-B, when F_MgCl2·6H2O_ exceeds 50 wt%, T_melt_ and △H decrease significantly. The results show that there is an optimum mass fraction of MgCl_2_·6H_2_O for mixture-A/mixture-B. It can be seen from the experimental results (Figure 1, Figure 2a,c, Table 2) that the optimum addition of MgCl_2_·6H_2_O is between 20 wt% and 40 wt%. Therefore, mixtures-A/B containing 25 wt% and 35 wt% MgCl_2_·6H_2_O were prepared and characterized by DSC to determine the optimum F_MgCl2·6H2O_. As shown in Figure 2b,d, mixture-A and mixture-B containing 30 wt% exhibit excellent thermal properties (NH_4_Al(SO_4_)_2_·12H_2_O-30 wt% MgCl_2_·6H_2_O: T_melt_ = 64.15 °C, △H = 192.1 kJ/kg; KAl(SO_4_)_2_·12H_2_O −30 wt% MgCl_2_·6H_2_O: T_melt_ = 60.93 °C, △H = 198.1 kJ/kg), which are denoted as mixture-A and mixture-B for further research in this work, respectively.

### 2.2. Structure and Thermal Properties of the Mixtures

**Figure 3:**
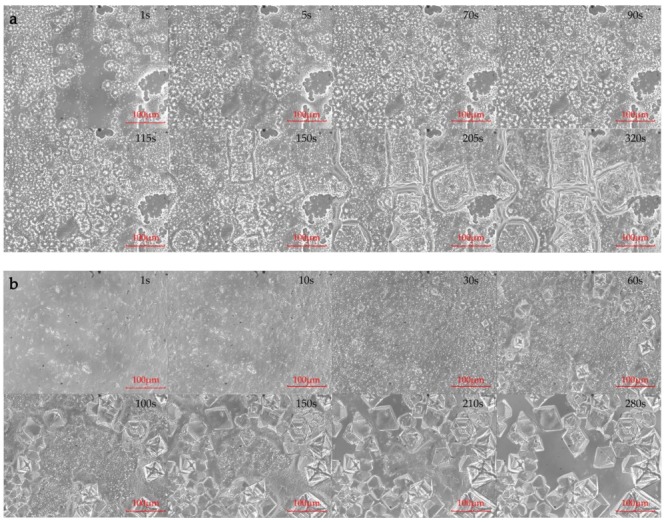
Polarized microscope images of (**a**) mixture-A and (**b**) mixture-B during crystallization.

Figure 3 displays the polarized microscope images obtained at different times during crystallization. The MgCl_2_·6H_2_O crystals are acicular, with sizes of 0.1–1 μm. On the other hand, NH_4_Al(SO_4_)_2_·12H_2_O and KAl(SO_4_)_2_·12H_2_O exhibit octahedral crystals with sizes of 100–150 μm, which are much larger than those of MgCl_2_·6H_2_O. In the first stage of the crystallization of mixture-A, a number of acicular crystals (MgCl_2_·6H_2_O) grow rapidly within 1 s. Some 70 s later, it can be seen that several octahedral crystals (NH_4_Al(SO_4_)_2_·12H_2_O) start to grow at a slow rate. Finally, the acicular crystals stick into the gaps among the octahedral crystals or attach to the surfaces of the octahedral crystals. As shown in the crystal image (Figure 3a, 320 s) of the mixture-A, two crystals with different shapes are tightly bound together to form the crystal of mixture A. Similarly, the acicular crystals (MgCl_2_·6H_2_O) grow rapidly during the crystallization of mixture-B. The octahedral crystals (KAl(SO_4_)_2_·12H_2_O) begin to appear at 30 s and develop gradually. It can be seen from Figure 3b that the acicular crystals finally attach to the surfaces of the octahedral crystals. However, there is no connection of acicular crystal between two octahedral crystals.

**Figure 4:**
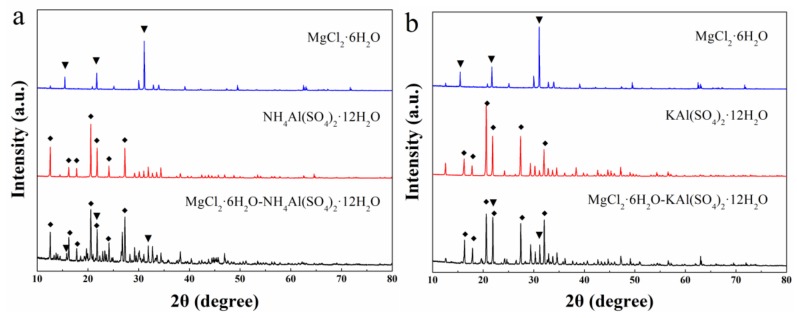
XRD patterns of MgCl_2_·6H_2_O, NH_4_Al(SO_4_)_2_·12H_2_O, KAl(SO_4_)_2_·12H_2_O and (**a**) mixture-A; (**b**) mixture-B.

Figure 4 displays the XRD patterns of MgCl_2_·6H_2_O, NH_4_Al(SO_4_)_2_·12H_2_O, KAl(SO_4_)_2_·12H_2_O, mixture-A (a) and mixture-B (b). It can be seen from Figure 4a that the XRD pattern of the mixture-A contains the diffraction peaks with strong intensities for NH_4_Al(SO_4_)_2_·12H_2_O and the ones with low intensities for MgCl_2_·6H_2_O. This is reasonable because the low content (30 wt%) of MgCl_2_·6H_2_O in the mixture and the much smaller sizes of the MgCl_2_·6H_2_O crystals comparing to those of NH_4_Al(SO_4_)_2_·12H_2_O (Figure 3). It is revealed that the mixture is composed of MgCl_2_·6H_2_O and NH_4_Al(SO_4_)_2_·12H_2_O. Moreover, several new diffraction peaks with low intensities appear in the XRD pattern of the mixture, which might originate from the integrated crystals between MgCl_2_·6H_2_O and NH_4_Al(SO_4_)_2_·12H_2_O. Different from Figure 4a, only the diffraction peaks of MgCl_2_·6H_2_O and KAl(SO_4_)_2_·12H_2_O are shown in the XRD pattern of mixture-B, and there is no new diffraction peak appears (Figure 4b). Therefore, the MgCl_2_·6H_2_O and KAl(SO_4_)_2_·12H_2_O are only physically mixed without any chemical reaction in the preparation of the mixture-B.

**Figure 5:**
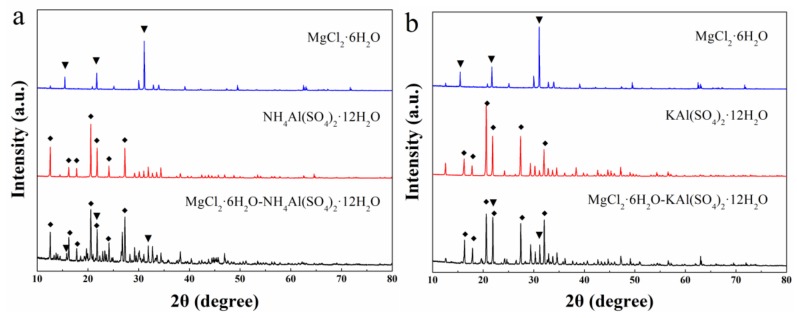
DSC curves of (**a**) MgCl_2_·6H_2_O, NH_4_Al(SO_4_)_2_·12H_2_O and mixture-A; (**b**) MgCl_2_·6H_2_O, KAl(SO_4_)_2_·12H_2_O and mixture-B.

Figure 5 shows the DSC melting curves of MgCl_2_·6H_2_O, NH_4_Al(SO_4_)_2_·12H_2_O, KAl(SO_4_)_2_·12H_2_O and mixtures. As shown in Figure 5a,b, the melting points are measured to be 64.15 °C and 60.93 °C for mixture-A and mixture-B, respectively. The melting points are much lower than the boiling point of water (100 °C, at standard atmospheric pressure). It can be inferred that the mixtures do not suffer from the instability of being prone to lose crystal water during melting. Furthermore, the melting enthalpy are 192.1 kJ/kg and 198.1 kJ/kg for mixture-A and mixture-B, respectively. Compared to the eutectic salt containing MgCl_2_·6H_2_O such as MgCl_2_·6H_2_O-Mg(NO_3_)_2_·6H_2_O and MgCl_2_·6H_2_O-CaCl_2_·6H_2_O [23,33,35,36], mixture-A and mixture-B have much larger melting enthalpy. The suitable melting points (64.15 °C, 60.93 °C) and the large latent heat (192.1 kJ/kg, 198.1 kJ/kg) of mixture-A and mixture-B demonstrate their great potential in for solar water heating systems.

**Figure 6:**
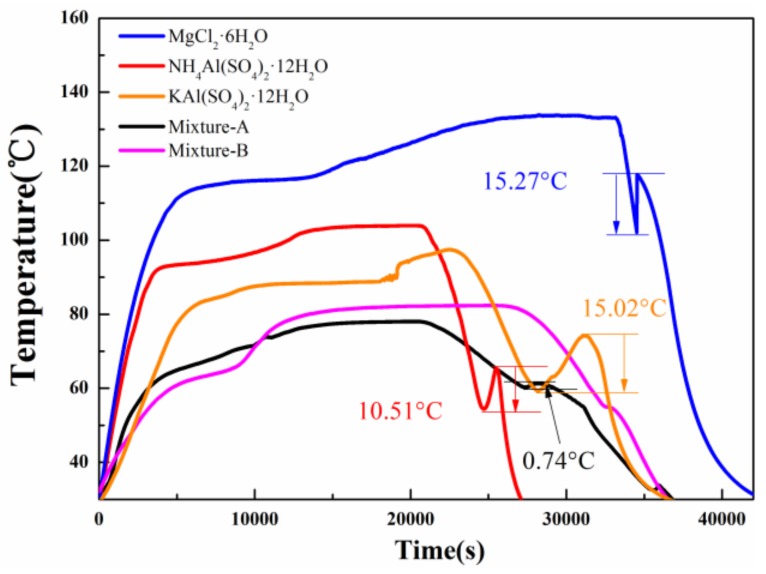
Cooling curves of three hydrated salts and mixture-A/B.

The cooling curves of MgCl_2_·6H_2_O, NH_4_Al(SO_4_)_2_·12H_2_O, KAl(SO_4_)_2_·12H_2_O and mixtures are displayed in Figure 6. The supercooling degree is found to be about 15.27 °C for MgCl_2_·6H_2_O, 10.51 °C for NH_4_Al(SO_4_)_2_·12H_2_O and 15.02 °C for KAl(SO_4_)_2_·12H_2_O. However, mixture-A exhibits a supercooling degree of 0.74 °C and mixture-B has no supercooling degree. The experimental results show that the combination of two hydrated salts (MgCl_2_·6H_2_O and NH_4_Al(SO_4_)_2_·12H_2_O or KAl(SO_4_)_2_·12H_2_O) lead to a low supercooling degree for the mixture. Compared to MgCl_2_·6H_2_O, mixture-A/mixture-B has a more suitable phase change temperature, higher melting enthalpy and lower supercooling degree. On the other hand, although the latent heat of NH_4_Al(SO_4_)_2_·12H_2_O or KAl(SO_4_)_2_·12H_2_O is higher than that of mixture-A/mixture-B, the large supercooling degree greatly limits its practical applications. These results suggest that mixture-A and mixture-B show greater promise for practical applications as compared with MgCl_2_·6H_2_O, NH_4_Al(SO_4_)_2_·12H_2_O and KAl(SO_4_)_2_·12H_2_O.

**Figure 7:**
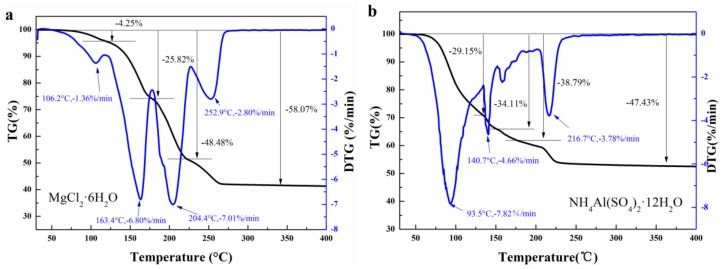

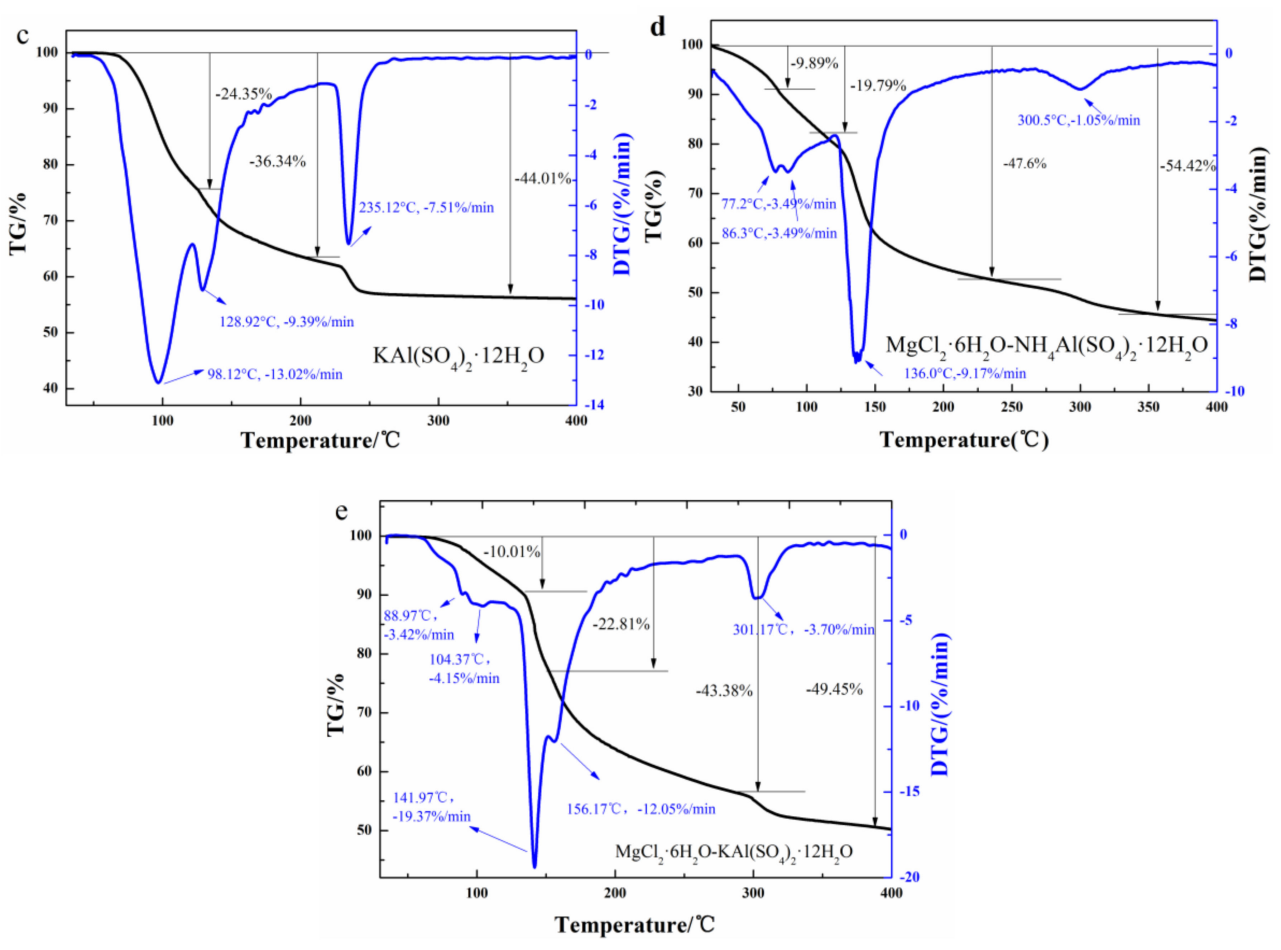
TGA curves of (**a**) MgCl_2_·6H_2_O, (**b**) NH_4_Al(SO_4_)_2_·12H_2_O, (**c**) KAl(SO_4_)_2_·12H_2_O, (**d**) mixture-A and (**e**) mixture-B.

The thermal stability of the mixture was evaluated by TGA. Figure 7 illustrates the weight loss curves of three hydrated salts and mixture-A/B. For MgCl_2_·6H_2_O (Figure 7a), it starts to decompose at about 60 °C and the weight loss due to the adsorbed air humidity is about 4.25%. At the temperature of 163.4 °C, the weight loss reach the second peak and the weight loss at this stage is 24.64%. After that, the product MgCl_2_·4H_2_O starts to decompose, resulting in the maximum rate of water loss. The MgCl_2_·4H_2_O lose two crystal waters and becomes MgCl_2_·2H_2_O. However, the weight loss of the sample is 25.82% at 179.81 °C, which is lower than the theoretical value of 35.42%. It indicates that the product at this stage is not only MgCl_2_·2H_2_O but also partially undecomposed MgCl_2_·4H_2_O. When the temperature increased to 289.23 °C, the sample decompose finally into a mixture of MgOHCl·H_2_O and MgO. The total weight loss of MgCl_2_·6H_2_O is 58.07%. Except for the first weight loss corresponding to adsorbed air humidity, the experimental value (58.07% − 4.25% = 53.82%) is coincide with the theoretical value of 6 crystal waters (53.2%). For NH_4_Al(SO_4_)_2_·12H_2_O (Figure 7b), the sample loses ten crystal waters in the first stage (93~175 °C) with the weight loss of 38.79 %. In the second stage (175~250 °C), the sample continues to lose two crystal waters and the final weight loss is 47.43%. According to the experimental result, the value of crystal waters lost from the sample is calculated to be 11.8, which is almost equivalent to 12. Similar to NH_4_Al(SO_4_)_2_·12H_2_O, KAl(SO_4_)_2_·12H_2_O loses nine crystal waters at 93.5~200 °C, as illustrated in Figure 7c. When the temperature continues to increase and exceeds 250 °C, the remaining three crystal waters are lost and the final weight loss is 44.01%. The error between experimental result and theoretic value (45.53%) is only 3.34%.

As shown in Figure 7d, mixture-A (MgCl_2_·6H_2_O-NH_4_Al(SO_4_)_2_·12H_2_O) has multiple weight loss steps, which are different from those of MgCl_2_·6H_2_O and NH_4_Al(SO_4_)_2_·12H_2_O. In particular, the mixture exhibits a lower onset decomposition temperature as compared to its two components because of its lower phase change temperature (64.15 °C). Moreover, a weight loss of 19.79% occurred for the mixture at the temperature of 143.23 °C. The experimental results suggest that the thermal property of mixture-A is different from those of MgCl_2_·6H_2_O and NH_4_Al(SO_4_)_2_·12H_2_O, verifying the formation of the mixture between two components. It can be seen from Figure 7e that mixture-B has a similar process of weight loss as mixture-A. And the final weight loss (49.45%) of mixture-B (Figure 7e, MgCl_2_·6H_2_O-KAl(SO_4_)_2_·12H_2_O) is between those of MgCl_2_·6H_2_O (58.07%) and KAl(SO_4_)_2_·12H_2_O (44.01%).

### 2.3. Thermal Reliability of Mixture-A and Mixture-B

**Figure 8:**
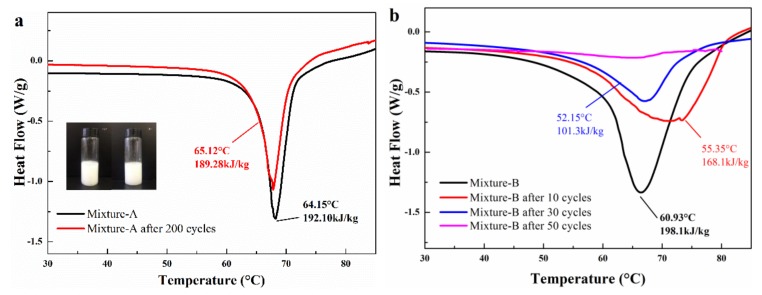
DSC result of (**a**) the mixture-A and (**b**) mixture-B before and after tests.

**Figure 9:**
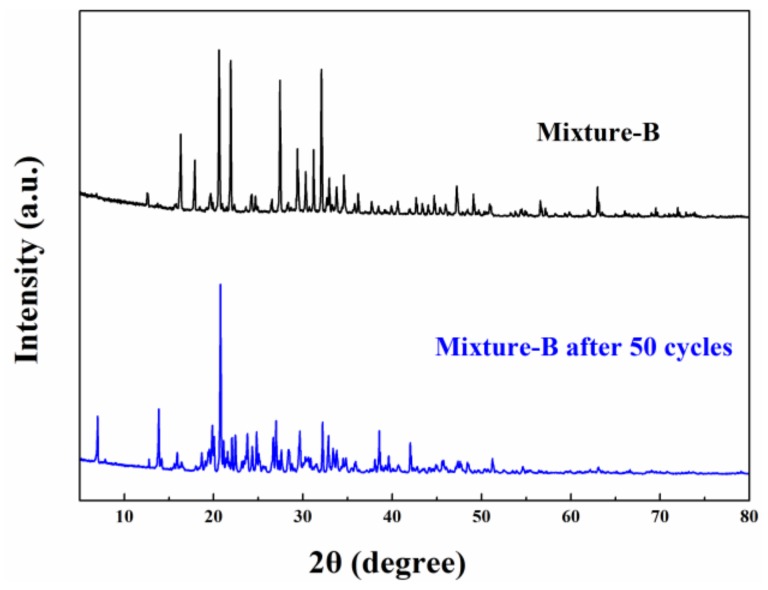
XRD patterns of Mixture-B before and after experiencing 50 heating-cooling cycles.

As we know, it is essential that PCM has good thermal reliability to undergo a number of thermal cycles. Therefore, the melting-freezing cycle test was carried out on mixture-A and mixture-B to investigate their thermal reliabilities. Figure 8 shows the DSC result of the mixture-A and mixture-B before and after experiencing different numbers of melting-freezing cycle tests. There is significant difference in thermal reliability between mixture-A and mixture-B. After 200 cycles of the melting-freezing cycle test, mixture-A exhibits a phase change temperature of 65.12 °C and a melting enthalpy of 189.28 kJ/kg (Figure 8a). Compared with the experimental result before the test, the deviations in phase change temperature and melting enthalpy are only 1.51% and 1.20%, respectively. However, the thermal property of mixture-B changes greatly with the increase in the number of cycles. As shown in Figure 8b, the melting enthalpy of mixture-B decreases to 101.3 kJ/kg after undergoing 30 cycles melting-freezing cycle tests. As mentioned in Section 2.2. Structure and thermal properties of the mixtures, the acicular crystals (MgCl_2_·6H_2_O) only stick to the surface of the octahedral crystal (KAl(SO_4_)_2_·12H_2_O) while not connect two octahedral crystals. The lack of tight connection between the two crystals results in poor thermal reliability of mixture-B. Furthermore, the structure of mixture-B has changed after the cycling tests, as shown in Figure 9. The significant weakening of most of the diffraction peaks may be due to the reduction of KAl(SO_4_)_2_·12H_2_O in mixture-B. In fact, NH_4_Al(SO_4_)_2_·12H_2_O and KAl(SO_4_)_2_·12H_2_O exhibit different thermal reliability although they have similar structures and properties. As shown in Table 3, the melting-freezing cycle tests of NH_4_Al(SO_4_)_2_·12H_2_O and KAl(SO_4_)_2_·12H_2_O were carried out. It is found that the melting enthalpy of KAl(SO_4_)_2_·12H_2_O was reduced by more than 50% after 10 cycles of 60–105 °C. The large decrease in melting enthalpy may be because the poor reversibility of the combination of aluminum potassium sulfate and crystal waters. In this work, when the number of cycles reaches 10, the melting enthalpy of mixture-B decreases by 15.14% compared to the sample before the cycle test. It can be known that the combination of MgCl_2_·6H_2_O and KAl(SO_4_)_2_·12H_2_O can improves the thermal reliability of KAl(SO_4_)_2_·12H_2_O. Nevertheless, mixture-B can’t conform to requirements because the PCM is expected to be reused many times to reduce costs while 10 cycles are not enough. By comparison, the mixture-A with excellent thermal reliability is a promising material for practical applications.

**Figure 10:**
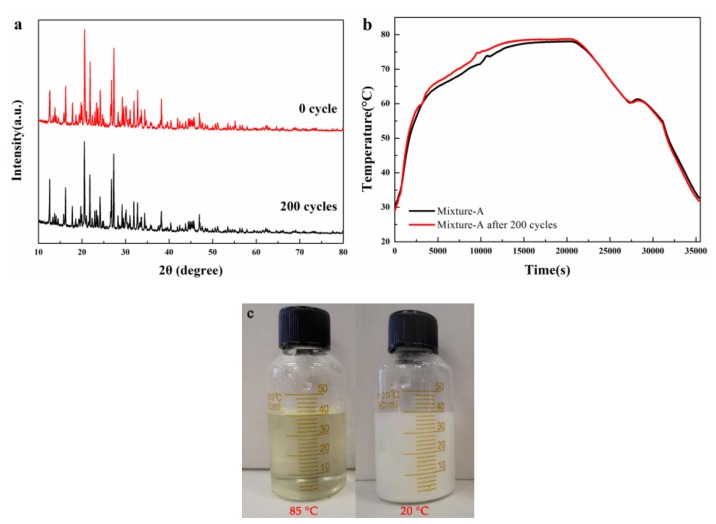
XRD patterns (**a**) and cooling curves (**b**) of the mixture-A before and after experiencing 200 heating-cooling cycles; and the material expansion test of mixture-A (**c**).

In order to ensure that the structure of the mixture-A didn’t change after the cycle tests, XRD patterns and cooling curves before and after experiencing 200 melting-freezing cycles were measured. It can be seen from Figure 10a that the XRD patterns before and after the tests are almost the same, suggesting that the mixture-A has good structure stability. Moreover, the cooling curves of the mixture-A before and after the test almost coincide with each other (Figure 10b). It is revealed that the MgCl_2_·6H_2_O-NH_4_Al(SO_4_)_2_·12H_2_O mixture possesses excellent thermal reliability, making it show great potentials for practical applications. In addition, the material expansion experiment result shows that the mixture A has no significant volume change before and after the phase change (Figure 10c).

## 3. Materials and Methods

### 3.1. Materials and Reagents

Magnesium chloride hexahydrate (MgCl_2_·6H_2_O, AR), aluminum ammonium sulfate dodecahydrate (NH_4_Al(SO_4_)_2_·12H_2_O, AR) and aluminum potassium sulfate dodecahydrate (KAl(SO_4_)_2_·12H_2_O, AR) were purchased from Guangzhou Chemical Reagent Factory (Guangzhou, China).

### 3.2. Sample Preparation

#### 3.2.1. MgCl_2_·6H_2_O-NH_4_Al(SO_4_)_2_·12H_2_O Mixtures

A series of MgCl_2_·6H_2_O-NH_4_Al(SO_4_)_2_·12H_2_O mixtures with different mass fractions of MgCl_2_·6H_2_O were prepared for determining the mixture composition of MgCl_2_·6H_2_O and NH_4_Al(SO_4_)_2_·12H_2_O. The experimental steps are as follows: Firstly, the MgCl_2_·6H_2_O and NH_4_Al(SO_4_)_2_·12H_2_O were weighed and placed in a reagent bottle. After simply stirring the two hydrated salts, the mixture was heated to 85 °C and maintained for 3 h. Then, intermittent stirring was used to ensure uniform mixing of the two hydrated salts during heating process. Finally, the solution was cooled at room temperature to obtain a MgCl_2_·6H_2_O-NH_4_Al(SO_4_)_2_·12H_2_O mixture.

#### 3.2.2. MgCl_2_·6H_2_O-KAl(SO_4_)_2_·12H_2_O Mixtures

In order to determine the optimum content of MgCl_2_·6H_2_O, a series of mixtures with different proportions of MgCl_2_·6H_2_O and KAl(SO_4_)_2_·12H_2_O were prepared. The experimental steps are same as that for preparing MgCl_2_·6H_2_O-NH_4_Al(SO_4_)_2_·12H_2_O mixture.

### 3.3. Characterization

The phase change temperature and latent heat of the samples were measured using a differential scanning calorimeter (DSC, DSC Q20, TA Instruments, New Castle, DE, USA). For DSC measurements, 5–10 mg for every sample was sealed in an aluminum pan and heated from 30 °C to 100 °C at a heating rate of 5 °C/min under a constant stream of nitrogen at a flow rate of 50 mL/min.

The structure of the sample was characterized by an X-ray diffraction (XRD, D8-ADVANCE, Bruker, Bruker, Billerica, MA, USA) using Cu Kα radiation (λ = 1.5406 Å). Diffraction patterns of the mixture were collected in the 2θ ranges from 10° to 80°.

The changes in morphology and microstructure the sample during crystallization were observed by using a polarization microscope (Observer.A1, Zeiss, Oberkochen, Germany). 30–50 mg of each sample was put on the glass slide to experience a temperature change from 90 °C to 25 °C.

In order to investigate the thermal stability of the mixture, the thermal gravimetric analysis of the samples were conducted by a thermoanalyzer instrument (TGA, STA409PC, Netzsch, Waldkraiburg, Germany). Each sample (8–12 mg) was placed in an alumina crucible and then heated from 30 to 400 °C at a heating rate of 10 °C/min under a constant stream of nitrogen at a flow rate of 100 mL/min.

The thermal reliability of the mixture was tested by using the High-low temperature (alternate) humid heat test chamber (TH300, Shanghai Yiheng Scientific Instrument Co., Ltd., Shanghai, China). Before the melting-freezing cycle test, the mixture was sealed in a reagent bottle to avoid water loss. To ensure the reproducibility of experimental result, five samples (20~25 mg) were prepared for testing under the same cycle test conditions. The melting-freezing cycle experiment was carried out as following process: At first, the chamber was heated from room temperature to 80 °C at a rate of 5 °C/min. Then, the five samples were heated at 80 °C for 60 min to completely melt the mixture. After that, the chamber temperature decreased to 30 °C at a rate of 5 °C/min. Finally, the samples completely solidified after cooling 60 min at 30 °C. After repeating 200 cycles melting-freezing experiments, the samples were characterized by DSC and XRD to study the thermal reliability.

### 3.4. Measurement of Cooling Curves

**Figure 11:**
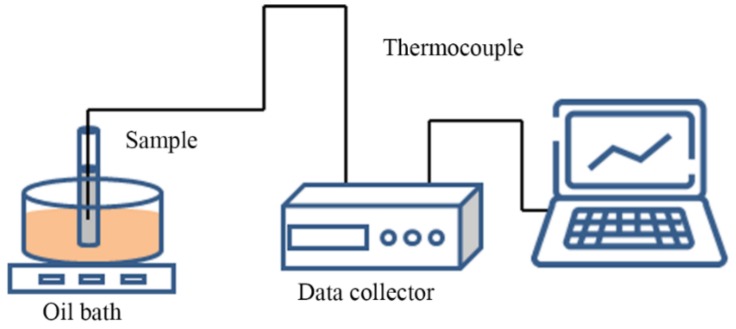
Schematic diagram of experimental setup for testing cooling curves.

As introduced in Section 3.3. Characterization, the mass of every DSC sample was controlled at 5ߝ10 mg, which was much less than the mass of PCM used in the industry. Note that the sample amount, heating rate, sample compositions would interfere in the DSC signal [37,38,39]. In order to avoid the influence of small sample amount on PCM solidification temperature measurement, the measurement of T-history or cooling curve was widely used to determine the supercooling degree of PCM samples [40,41]. Therefore, the crystallization properties of the mixture samples in this work were characterized by measuring their cooling curves. Figure 11 shows a schematic diagram of the experimental setup, which consists of a thermostatic silicone oil bath box and a thermal energy storage (TES) unit. The silicone-oil bath could control the temperature automatically with an accuracy of ±0.01 °C. And the TES unit used a cylindrical glass container with an inner diameter of 25 mm, a wall thickness of 1 mm and a length of 200 mm to place the sample. As shown in Figure 11, a K-type thermocouple was placed at the center of the cylindrical glass container to monitor the temperature change of each sample with a measurement error of ±0.1°C. Moreover, the test points were at the same depth (20 mm away from bottom of the container) in different tests, to make sure the effects of input heat flux from top and bottom on them were identical. On the other hand, two-third of the volume of the container was filled with the test sample, and the remaining space was left to adapt to the thermal expansion of the PCM. During the measurement, the TES unit was put into the oil bath and heated/cooled with the following program. Firstly, the oil was heated at a rate of 1 °C/min from the room temperature to the specified temperature (80 °C for mixtures, 105 °C for NH_4_Al(SO_4_)_2_·12H_2_O/KAl(SO_4_)_2_·12H_2_O and 135 °C for MgCl_2_·6H_2_O). Secondly, the thermostatic silicone oil bath box was maintained at the specified temperature for 2 h until the samples completely melted. Finally, the oil was cooled down to 30 °C at a rate of 1 °C/min. At the same time, temperature data in the experiment was collected using a data logger (Agilent 34970A, Santa Clara, CA, USA).

## 4. Conclusions

Two series of mixtures (mixture-A: MgCl_2_·6H_2_O-NH_4_Al(SO_4_)_2_·12H_2_O and mixture-B: MgCl_2_·6H_2_O-KAl(SO_4_)_2_·12H_2_O) were investigated as PCMs for solar water heating system. The thermal property, structure and the thermal stability of PCM were studied systematically. The experimental results can be concluded as follows:

1) The mass fraction of MgCl_2_·6H_2_O in mixture-A or mixture-B is determined to be 30 wt%. It is found that the mixture-A has a melting point of 64.15 °C, a melting enthalpy of 192.1 kJ/kg, and a very low supercooling degree (0.74 °C). On the other hand, the melting point and melting enthalpy of mixture-B without supercooling degree are 60.93 °C and 198.1 kJ/kg, respectively.
The XRD pattern of mixture-A shows that MgCl_2_·6H_2_O and NH_4_Al(SO_4_)_2_·12H_2_O combined physically and have no chemical reaction, like mixture-B (MgCl_2_·6H_2_O and KAl(SO_4_)_2_·12H_2_O).
The polarized microscope images show that the crystallization behaviors of mixture-A and mixture-B. The acicular crystals (MgCl_2_·6H_2_O) not only attach on the surface of the octahedral crystals (NH_4_Al(SO_4_)_2_·12H_2_O) but also stick into the gaps between octahedral crystals. Furthermore, the free acicular crystals (MgCl_2_·6H_2_O) in the mixture-B eventually only adhere to the surface of the octahedral crystals (KAl(SO_4_)_2_·12H_2_O).
The 200 heating-cooling cycle test reveals that the mixture-A possesses good structure stability and excellent thermal reliability while the mixture-B has poor thermal reliability. The suitable phase change temperature, high latent heat along with excellent thermal reliability make the MgCl_2_·6H_2_O-NH_4_Al(SO_4_)_2_·12H_2_O mixture show great potential in solar water heating systems.
In conclusion, with a suitable melting point, high latent heat, good structure stability and excellent thermal reliability, mixture-A is a highly promising thermal energy storage phase change material for SWH system. In future work, the heat transfer efficiency of mixture-A and its compatibility with devices will require further research and exploration to better apply materials to practical SWH system or other thermal storage system.

## Figures and Tables

**Table 1:** Different mixture series.

Mixture	Compositions	
A	MgCl_2_·6H_2_O	NH_4_Al(SO_4_)_2_·12H_2_O
B	MgCl_2_·6H_2_O	KAl(SO_4_)_2_·12H_2_O

**Table 2:** Thermal characteristics of mixtures (mixture-A and mixture-B) with different mass fractions of MgCl_2_·6H_2_O below 85 °C.

Mixture-A			Mixture-B		
F_MgCl2·6H2O_^1^ (wt%)	T_melt_ (°C) ^2^	△H (kJ/kg) ^3^	F_MgCl2·6H2O_ (wt%)	T_melt_ (°C)	△H (kJ/kg)
10	63.54	141.8	10	61.00	165.0
20	63.90	131.9	20	61.89	173.6
30	64.15	192.1	30	60.93	198.1
40	65.08	138.4	40	64.67	173.3
50	65.97	83.17	50	67.92	145.6
60	53.90	60.13	60	60.86	100.0
70	52.23	46.59	70	50.42	92.14
80	51.94	45.72	80	45.49	57.30
90	48.00	7.747	90	49.00	24.21

^1^ F_MgCl2·6H2O_—the mass fraction of MgCl_2_·6H_2_O; ^2^ T_melt_—the melting point of mixture; ^3^ ΔH—the melting enthalpy of mixture.

**Table 3:** Thermal reliability of NH_4_Al(SO_4_)_2_·12H_2_O and KAl(SO_4_)_2_·12H_2_O.

	NH_4_Al(SO_4_)_2_·12H_2_O		KAl(SO_4_)_2_·12H_2_O	
	T_melt_ (°C)	△H (kJ/kg)	T_melt_ (°C)	△H (kJ/kg)
0 cycles	93.86	264.0	90.85	261.3
After 5 cycles 60–105 °C	93.21	263.2	90.31	185.9
After 10 cycles 60–105 °C	93.50	251.0	91.83	107.3
After 20 cycles 60–105 °C	93.37	218.9	90.79	104.5
